# Thyroid Carcinoma with *NSD3::NUTM1* Fusion: a Case with Thyrocyte Differentiation and Colloid Production

**DOI:** 10.1007/s12022-021-09700-2

**Published:** 2022-01-08

**Authors:** Derek B. Allison, Justin Rueckert, Virgilius Cornea, Cortney Y. Lee, Julie Dueber, Therese Bocklage

**Affiliations:** 1 Department of Pathology and Laboratory Medicine, University of Kentucky College of Medicine, Lexington, KY USA; 2 Department of Surgery, Division of Endocrine Surgery, University of Kentucky College of Medicine, Lexington, KY USA

**Keywords:** Thyroid carcinoma, NUT carcinoma, *NSD3*, *NSD3::NUTM1*, *BRD4*, *NUTM1*

## Abstract

In this report, we present a high-grade thyroid carcinoma with an *NSD3::NUTM1* fusion detected on expanded next-generation sequencing testing. Nuclear protein of the testis (NUT) carcinomas comprise high-grade, aggressive tumors characterized by rearrangements of the *NUTM1* gene with various partner genes, most commonly the bromodomain protein genes *BRD4* and *BRD3*. Approximately 10% of NUT carcinomas contain an *NSD3::NUTM1* fusion. NUT carcinomas manifest as poorly differentiated or undifferentiated squamous carcinomas, and 33% show areas of mature squamous differentiation. Only exceptionally have NUT carcinomas shown histology discordant from poorly differentiated/undifferentiated squamous carcinoma, and a thyroid NUT carcinoma with histologic thyrocyte differentiation has not been described to date. Our patient’s tumor exhibited mixed cytologic features suggestive of squamoid cells or papillary thyroid carcinoma cells. Overt squamous differentiation was absent, and the tumor produced colloid in poorly formed follicles. Immunophenotypically, the carcinoma was consistent with thyrocyte differentiation with expression of monoclonal PAX8, TTF1, and thyroglobulin (the last predominantly in extracellular colloid). There was zero to < 2% reactivity for proteins typically diffusely expressed in NUT carcinoma: p40, p63, and cytokeratins 5/6. NUT protein expression was equivocal, but fluorescence in situ hybridization confirmed a *NUTM1* rearrangement. This exceptional case suggests that *NUTM1* fusions may occur in an unknown number of aggressive thyroid carcinomas, possibly with distinctive histologic features but with thyrocyte differentiation. Recognition of this entity potentially has significant prognostic implications. Moreover, thyroid carcinomas with *NUTM1* fusions may be amenable to treatment with NUT carcinoma-targeted therapy such as a bromodomain and extraterminal domain protein small molecular inhibitor (BETi).

## Introduction 

Nuclear protein of the testis (NUT) carcinoma (previously termed NUT midline carcinoma) is an aggressive carcinoma that was first described in single case reports beginning in 1991 with the description of an aggressive thymic carcinoma showing a gene translocation, t(15;19)(q15;p13) [1]. Subsequently, a joint Japanese and American team characterized NUT carcinoma as a distinct entity in 2003 [2]. To date, approximately 200 cases of NUT carcinoma have been reported [3]. The typical NUT carcinoma harbors *BRD4*, *BRD3*, *NSD3*, or *Z4* bromodomain complex (BDC) protein genes as fusion partners to *NUTM1* and manifests histologically as an undifferentiated to poorly differentiated carcinoma [4]. Keratinization is detected in approximately one-third of cases [4]. Thus, NUT carcinoma has been considered a variant of high-grade squamous carcinoma.

The initial patients reported to have NUT carcinoma were usually children to young adults with a midline tumor. However, the name, “NUT midline carcinoma,” was replaced by “NUT carcinoma,” as more patients with non-midline tumors were identified. The additional involved reported sites include the lung, salivary glands, pancreas, kidney, adrenal gland, and bladder [4, 5]. Recently, three cases of NUT carcinoma were described as arising in the thyroid gland [6–8]. Two of the three cases showed characteristic histologic features of NUT carcinoma, of which one also expressed PAX8 in addition to diffuse p63 by immunohistochemistry [6, 7]. The third case lacks a detailed description but was diagnosed as an anaplastic thyroid carcinoma [8].

Herein, we present a primary thyroid carcinoma with features of thyrocyte differentiation including colloid production and nuclear features partially emulating papillary thyroid carcinoma. We will describe the cytologic and histologic findings, the immunohistochemical features, and the molecular genetic results. We will compare our patient’s case with the three other reported cases of *NUTM1* fusion carcinomas arising in the thyroid.

## Materials and Methods

### Review of Cytological and Histological Materials

The cytologic samples were obtained by ultrasound-guided fine-needle aspiration (FNA) of the primary thyroid tumor and intra-operative smears of the resected tumor. The cytopathologist co-authors reviewed the cytology smears and cell block sections. Papanicolaou, DiffQuik™, and rehydrated hematoxylin and eosin smears were prepared in addition to a formalin-fixed paraffin-embedded (FFPE) cell block of FNA rinses.

The histologic specimen was reviewed by the pathologist co-authors and comprised hematoxylin and eosin–stained sections of tumor blocks and adjacent thyroid parenchyma and neck soft tissue. One pathologist manually counted nuclear grooves, mitotic figures, and individual necrotic cells by quantifying two foci of 500 tumor cells each in histologically viable tumor regions.

### Immunohistochemistry Methods

Immunohistochemical (IHC) staining was performed initially on de-stained Papanicolaou cytology smears and thereafter on two representative tumor sections from the resection specimen. All IHC staining except BRAF^V600e^ and NUT antibody staining was performed at the University of Kentucky Health Sciences Center Clinical Laboratory using a Roche automated platform (Ventana, Roche US diagnostic headquarters, Indianaopolis, IN) or Dako automated platform (Autostainer Link 48, Glostrup, Denmark) with validated commercial antibodies using appropriate positive and negative controls. NUT antibody staining was performed by two commercial laboratories, Mayo Clinic Laboratories (Rochester, MN) and ARUP Laboratories (Salt Lake City, UT), and the slides including positive controls were reviewed by the study pathologists. Specific manual counts of percent reactivity for Ki67, p63, and p40 were performed by one study pathologist on 10 consecutive fields of 100 tumor cells each.

### Genetic Analysis Methods

Two dedicated FNA passes were performed and directly rinsed into a Veracyte FNAprotect® collection tube and sent for molecular analysis by the Afirma Xpression Atlas (XA) (Veracyte, South San Francisco, CA). This panel includes coverage of 905 genomic variants and 235 fusion pairs from 593 genes using targeted DNA sequencing and transcriptome RNA sequencing, as previously described. Notably, *NUTM1*, *NSD3*, *BRD3*, and *BRD4* fusions are not covered in the XA test (Afirma website, https://www.afirma.com).

FFPE tissue from the thyroidectomy specimen was sent for molecular analysis utilizing the Caris Molecular Intelligence Profile platform (Caris Life Sciences, Phoenix, AZ). This mutational analysis by next-generation sequencing (NGS) was performed on FFPE tissue samples using the Illumina NovaSeq 6000 sequencer (Illumina, Inc., San Diego, CA). Over 700 clinically relevant genes were analyzed with high coverage and a high read-depth; a separate panel was designed to enrich for over 20,000 genes at a lower depth. In addition, gene amplification/deletion was analyzed utilizing a 500-Mb single nucleotide polymorphisms panel (Agilent Technologies, Santa Clara, CA). In summary, this panel is validated to detect variant sequences, copy number alterations, tumor mutational burden (TMB), loss of heterozygosity (LOH), and microsatellite instability. Gene fusion and transcript variant detection by RNA sequencing were performed on mRNA isolated from FFPE tissue using the Agilent SureSelectXT Low Input Library prep chemistry utilizing the SureSelect Human All Exon V7 bait panel and the Illumina NovaSeq, allowing the detection of transcript variants and novel gene breakpoints.

Fluorescence in situ hybridization was performed by Mayo Clinic Laboratories (Rochester, Minnesota) using a *NUTM1* break-apart probe set (15q14; 5′*NUTM1*, 3′*NUTM1*) as a validated laboratory developed test. The assay was performed on a representative formalin-fixed, paraffin-embedded tissue section.

## Case Description and Results

### Clinical Presentation, Treatment, and Follow-Up

**Fig. 1:**
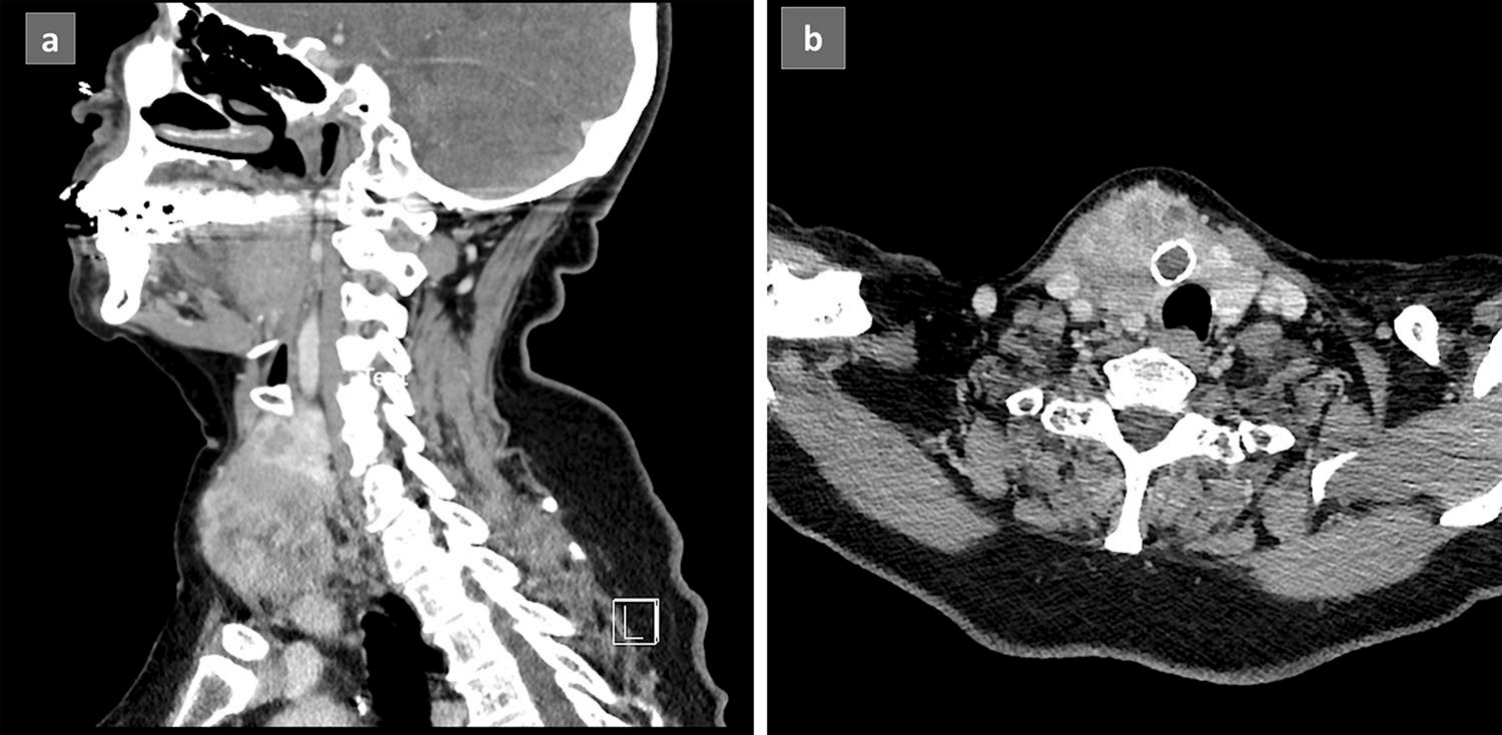
Computerized tomography (CT) of the 4.5 × 4.5 × 5.5-cm thyroid tumor: **a** sagittal and **b** coronal views showing a heterogeneous mass centered in the right lobe of the thyroid and extending into surrounding soft tissue causing mild leftward deviation of the trachea and larynx

A 72-year-old woman presented to her health care provider after she noted a new, asymptomatic neck mass. She had a 2-year history of hypothyroidism due to Hashimoto’s thyroiditis controlled on 25 mcg of levothyroxine. She denied radiation exposure and reported no family history of thyroid disease or thyroid cancer. On exam, a large visible and palpable, firm, fixed right thyroid nodule was noted. Ultrasound imaging of the mass revealed a large 4.5 × 4.6 × 4.9-cm solid, hypo- to isoechoic nodule in the right mid-inferior lobe extending into the isthmus causing tracheal deviation to the left. Serologic tests were within normal limits except for elevated thyroglobulin (42.2 ng/mL) and thyroglobulin antibodies (66.7 IU/mL). Computed tomography (CT) of the patient’s neck (Fig. 1a, b) and chest and a PET CT delineated the known mass without evidence of lymphadenopathy or distant disease.

The patient underwent surgical resection and central node dissection. Intraoperatively, the mass involved the right carotid sheath, but the vessel walls were uninvolved. The tumor was adherent to the anterior inferior trachea. Intraoperative frozen section revealed tumor abutting the entire margin on the thyroid specimen as expected. However, an edge of the remaining perichondrium was negative for tumor at frozen section. Both recurrent laryngeal nerves were identified and preserved intact, and both functioned satisfactorily via vagal stimulation after all dissection. Surgically, gross total resection was achieved. The patient recovered well from surgery and was released from the hospital on the fourth post-operative day. Three weeks after surgery, the patient’s serum thyroglobulin level had significantly declined to 2.0 ng/mL and serum thyroglobulin antibody was also lower at 28.1 IU/mL. The patient is currently undergoing adjuvant radioactive iodine ablation of remnant thyroid tissue.

### Fine Needle Aspiration Cytology Findings (Fig. 2a–d)

**Fig. 2:**
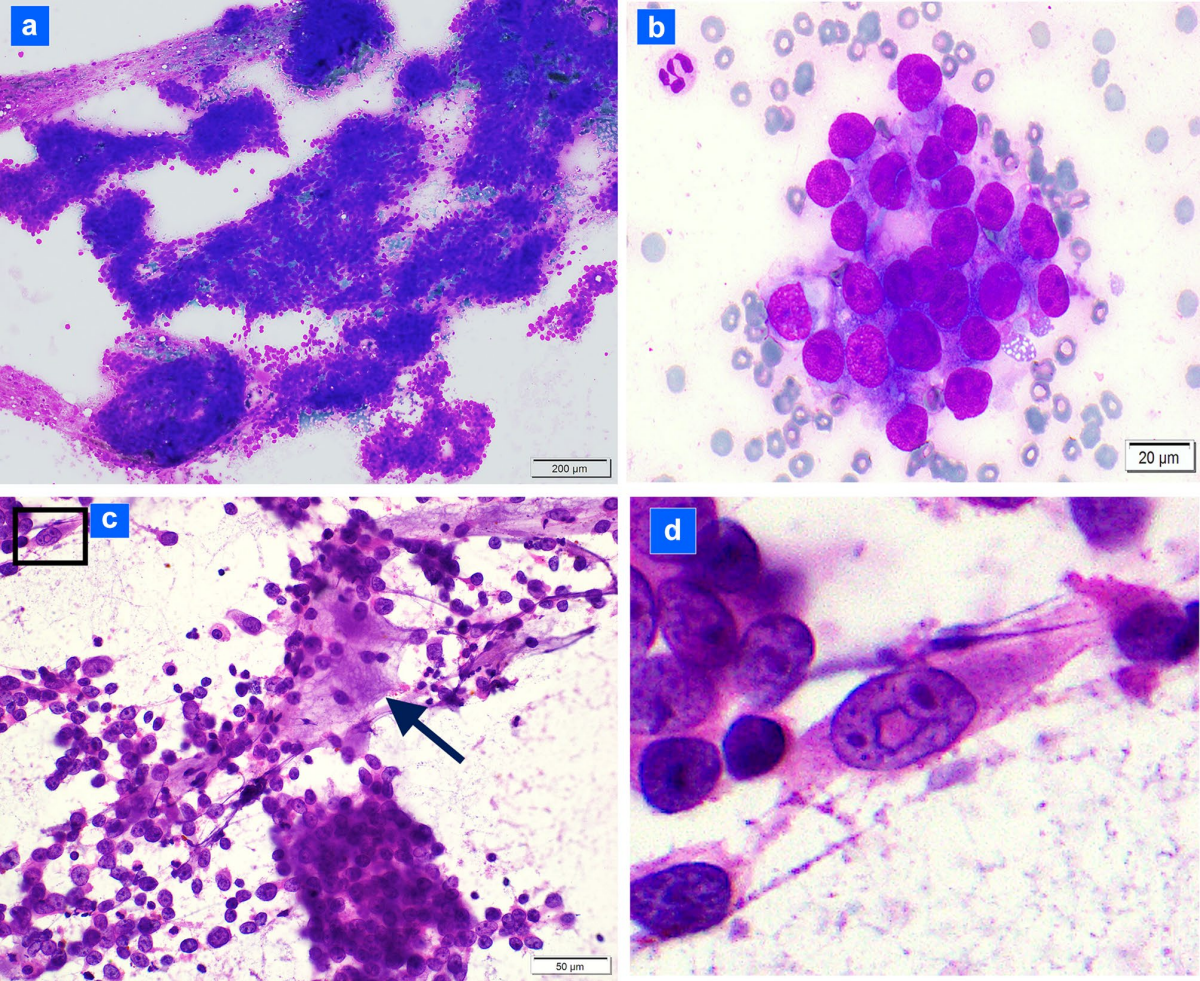
Cytologic features of the tumor: **a** on immediate smear, the tumor comprises cohesive groups of disorganized cells (DiffQuik™; 100 ×); **b** tumor cells exhibit a moderate to high nuclear to cytoplasmic ratio, round to oval nuclei and size ~ 3–5 times that of a red blood cell (DiffQuik™; 600 ×); **c** tumor cells exhibit prominent nucleoli with focal myxoid matrix (arrow) and rare nuclear pseudo-inclusion (box) (rehydrated hematoxylin and eosin immediate smear; 400 ×); **d** higher magnification of the tumor cell with a nuclear pseudo-inclusion (same cell seen in upper left of Fig. 2c; hematoxylin and eosin immediate smear; ~ 600 ×)

Direct smears from the fine needle aspiration biopsy of the thyroid tumor were abundantly cellular and consisted of cohesive clumps of unpolarized tumor cells (Fig. 2a). At higher magnification, the tumor cells appeared relatively monotonous with a moderate N/C ratio and round to oval nuclei with a few cells exhibiting mild nuclear contour irregularities (Fig. 2b). Most tumor nuclei contained a single prominent nucleolus. A few tumor nuclei exhibited nuclear grooves (Fig. 2c), and extremely rare nuclear pseudo-inclusions were present (Fig. 2d). Dense globules possibly comprising colloid and scant wispy myxoid material were intermixed within tumor cell groups (Fig. 2c). The background was necrotic to bloody. Two Papanicolaou-stained slides were de-stained to perform immunocytochemical staining. Tumor cells diffusely expressed AE1/AE3 cytokeratins and did not express CD45. A diagnosis of carcinoma was rendered noting a differential of papillary thyroid carcinoma, poorly differentiated thyroid carcinoma, and less likely, metastatic carcinoma.

### Resection Findings and Ancillary Testing Results

**Fig. 3:**
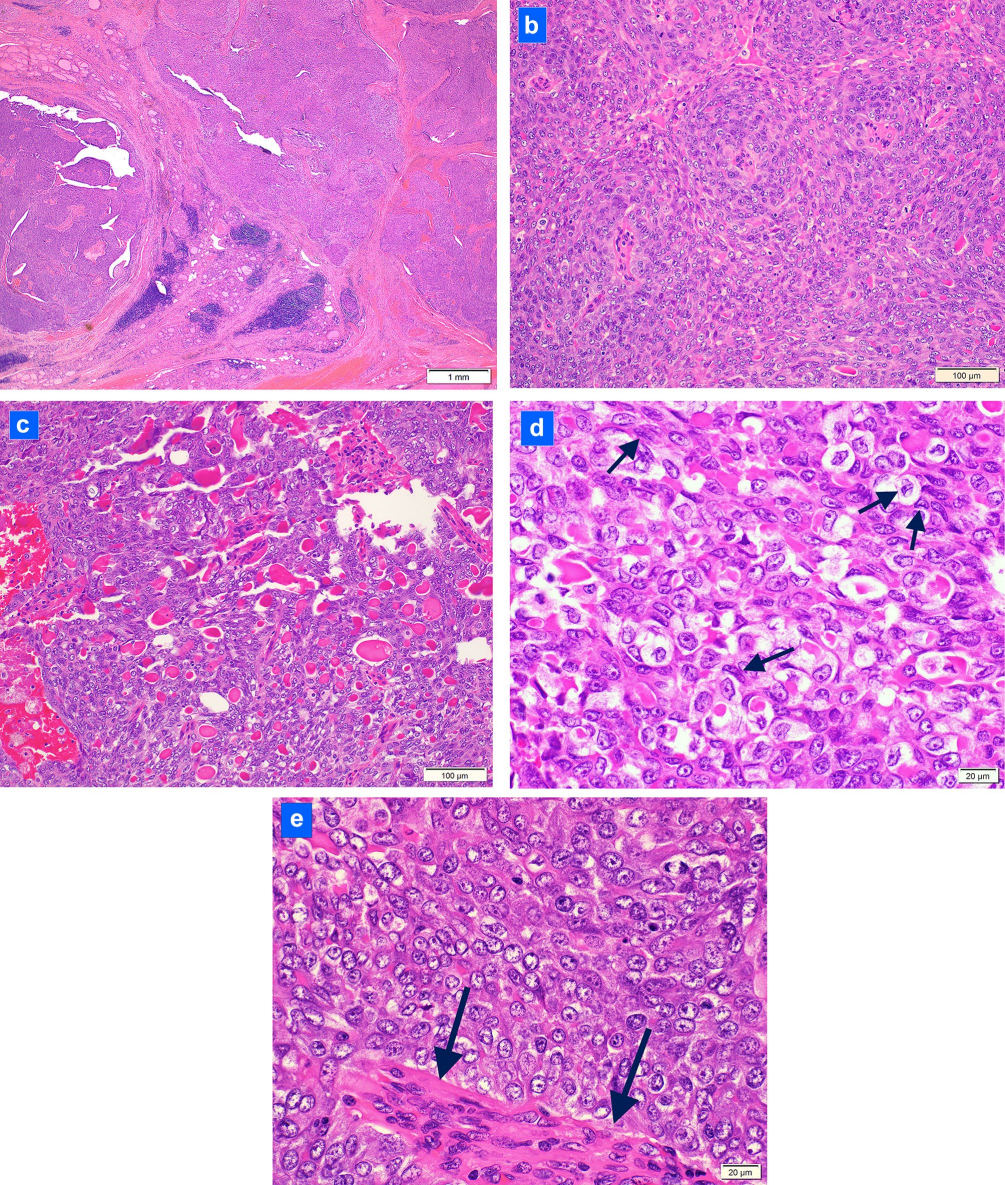
Histologic features of the tumor at resection: **a** the tumor forms multiple nodules in the thyroid (hematoxylin and eosin; 40 ×); **b** most of the tumor comprises nodules of tumor cells swirling around peculiar, distinctive, micro-proliferated blood vessels (hematoxylin and eosin; 200 ×); **c** numerous colloid globules intermixed among tumor cells (hematoxylin and eosin; 200 ×); **d** tumor cells exhibiting irregular nuclear contours, nuclear clearing and nuclear grooves (arrows) reminiscent of papillary thyroid carcinoma (hematoxylin and eosin; 600 ×); **e** tumor cells with nuclear clearing but no nuclear grooves and smooth nuclear contours and abnormal, micro-proliferated blood vessel (arrows) (hematoxylin and eosin; 600 ×)

Gross Findings (Fig. 3a–d): At resection, the 5-cm tumor involved the right and left lobes and isthmus and extended posteriorly and laterally into surrounding soft tissue. The tumor comprised closely apposed, soft, pink to red nodules with foci of necrosis and hemorrhage.

Microscopic Findings: The tumor replaced 70% of the thyroid and extended into perithyroidal soft tissue. At low power, the tumor formed solid nodules separated by fibrous septae (Fig. 3a). Within tumor nodules, cells were arranged as vague swirls around peculiar, micro-proliferated blood vessels reminiscent of the abnormal vessels of glioblastoma (Fig. 3b). Ten percent of the tumor exhibited a peritheliomatous growth pattern due to peripheral necrosis. Found in all tumor sections, small, dense eosinophilic globules consistent with colloid were interspersed among tumor cells. These foci were consistent with poorly formed follicles (Fig. 3c). Intracellular colloid was also identified. Wispy gray-blue myxoid matrix was focally present. The squamoid tumor cells were moderate in size (3–4 times the size of a lymphocyte) with a moderate N/C ratio, indistinct cell borders, and pale eosinophilic cytoplasm. Most tumor nuclei were oval to round featuring granular chromatin and typically one nucleolus. However, interspersed cells exhibited nuclear features suggestive of papillary thyroid carcinoma including irregular nuclear contours, nuclear clearing, and nuclear grooves (up to 7% of tumor cells) (Fig. 3d). Nuclear pseudo-inclusions were not identified. Mitoses averaged 8 per 10/hpf (up to 24 per 10/hpf). Individual necrotic cells were present averaging 1–2/hpf. Overt squamous differentiation, true papillae, and well-formed follicles were absent. Vascular invasion was present in one medium-sized vein. The residual non-involved thyroid showed multinodular hyperplasia, mild chronic lymphocytic thyroiditis, and one, 0.8-cm, acellular calcified nodule. A well-differentiated component such as papillary thyroid carcinoma or follicular carcinoma was not identified. Nine level VI lymph nodes were negative for carcinoma. The tumor was staged as pT3a/pN0 (following the AJCC Cancer Staging Manual, 8th ed). After review of the immunostaining panel results, and in light of the negative Afirma XPression Atlas™ results (see below), a diagnosis of “poorly differentiated thyroid carcinoma” was conferred along with a strong recommendation for broad panel next-generation sequencing testing due to the unusual histologic features and lack of typical molecular abnormalities.

### Immunohistochemistry Results (Table 1; Fig. 4a–f)

**Table 1:** Antibody details with staining results

**Antibody**	**Result**	**Clone**	**Manufacturer**
Cytokeratins AE1/AE3	Diffusely positive	AE1/AE3	DAKO
Cytokeratin 7	Diffusely positive	OV-TL 12/30	DAKO
Cytokeratins 5/6	Negative	D5/16 B4	DAKO
p63	Negative (< 2%)	4A4	Biocare
p40	Negative (< 2%)	BC28	Biocare
NUT	Equivocal: < 10% staining	Unknown	Unknown (performed at two external reference labs)
e-cadherin	Diffusely positive in tumor cell membranes	NCH-38	DAKO
PAX8	Diffusely positive	MRQ-50	Cell Marque/Ventana
TTF1	Diffusely positive	8G7G3/1	DAKO
Thyroglobulin	Positive in extracellular colloid and scattered tumor cells	Polyclonal Code IR509	DAKO
BRAF^V600E^	Negative	VE1	Ventana
BCL2	Patchy positive	124	DAKO
Ki67	30–53% positive	MIB-1	DAKO
S100 protein	Negative	Polyclonal Code IR504	DAKO
Mammaglobin	Negative	304-1A5	DAKO
CEA	Negative	IL-7	DAKO
Calcitonin	Negative	Polyclonal Code IR515	DAKO
Chromogranin	Negative	LK2H10	Cell Marque
CD5	Negative	4C7	DAKO
CD45	Negative	2B11 + PD7/26	DAKO

**Fig. 4:**
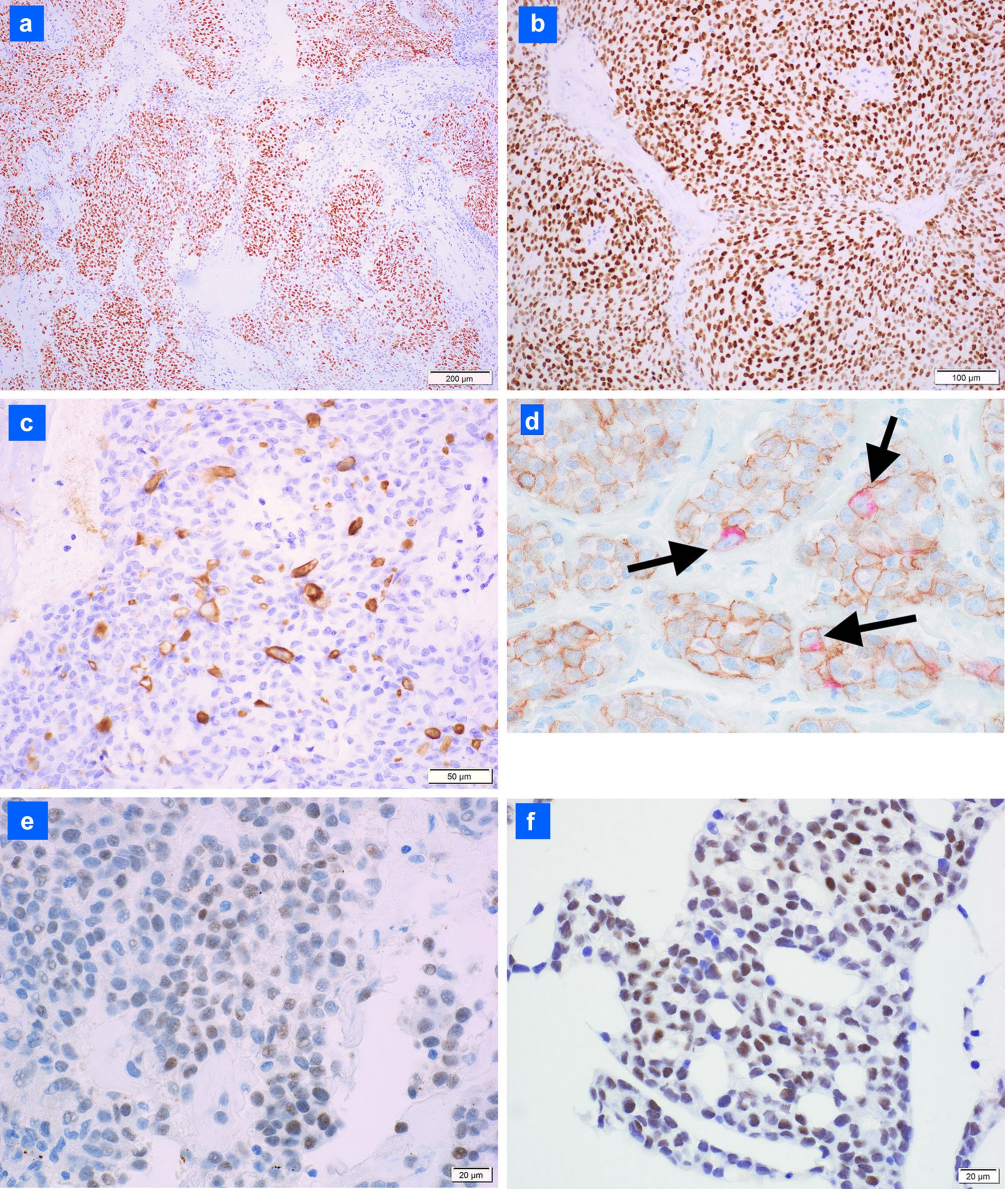
Selected immunohistochemical results (a brown chromogen is used for all antibodies with hematoxylin as a counter-stain except Fig. 4d in which a red chromogen is also used): **a** PAX8 is expressed in most tumor cells (100 ×); **b** TTF1 is strongly diffusely expressed (200 ×); **c** thyroglobulin antibody strongly decorates colloid globules (400 ×); **d** intracellular thyroglobulin is present in scattered tumor cells: e-cadherin (brown chromogen) outlines the cell membrane and surrounds thyroglobulin (red chromogen) (arrows; 600 ×); **e** focal (10% of tumor) equivocal weak NUT protein expression (Mayo Clinic Laboratories antibody; 600 ×); **f** focal (10% of tumor) NUT protein expression (ARUP Laboratories antibody; 600 ×)

#### Positive

Tumor cells diffusely expressed monoclonal PAX8 (Fig. 4a), TTF1 (Fig. 4b), and cytokeratin 7 with patchy expression of BCL2. Less than 2% of cells expressed p63 and p40. Thyroglobulin uniformly highlighted intra-tumoral interspersed eosinophilic globules confirming colloid composition (Fig. 4c). Dual IHC staining with e-cadherin and thyroglobulin also revealed scattered tumor cells containing intracellular, cytoplasmic thyroglobulin (Fig. 4d). Weak focal expression of NUT antibody in 10% of tumor cells (tested twice with antibodies from two commercial laboratories) was interpreted as equivocal (Fig. 4e, f). The proliferation index measured by Ki67 expression ranged from 30 to 53% (from manual counts of cold and hot spots of reactivity, respectively).

#### Negative

There was no expression of BRAF^V600e^ protein, cytokeratins 5/6, calcitonin, chromogranin, CEA, CD5, S-100 protein, and mammaglobin. Table 1 lists the antibodies, clone, manufacturer, and results.

### Genetic Testing

#### Afirma Xpression Atlas™ Results

 The Afirma XA test detected no gene variants or fusions.

#### CARIS MI Profile™ Results

 NGS sequencing revealed no pathogenic variant mutations in *BRAF*, *NRAS*, *RET*, *TP53*, or any other relevant cancer genes. The tumor was microsatellite stable, displayed a low TMB (1), and contained a low level of genomic LOH (7% of tested genomic segments exhibited LOH; assay threshold ≥ 16). RNA sequencing showed an *NSD3::NUTM1* gene fusion at exon 7: exon 3 splice site (transcript ID NM_017778.2/NM_175741.2). Fusions for *BRAF*, *NTRK1*/2/3, *RET*, and other relevant genes were not detected.

#### Mayo Clinic NUTM1 FISH Test Result

**Fig. 5:**
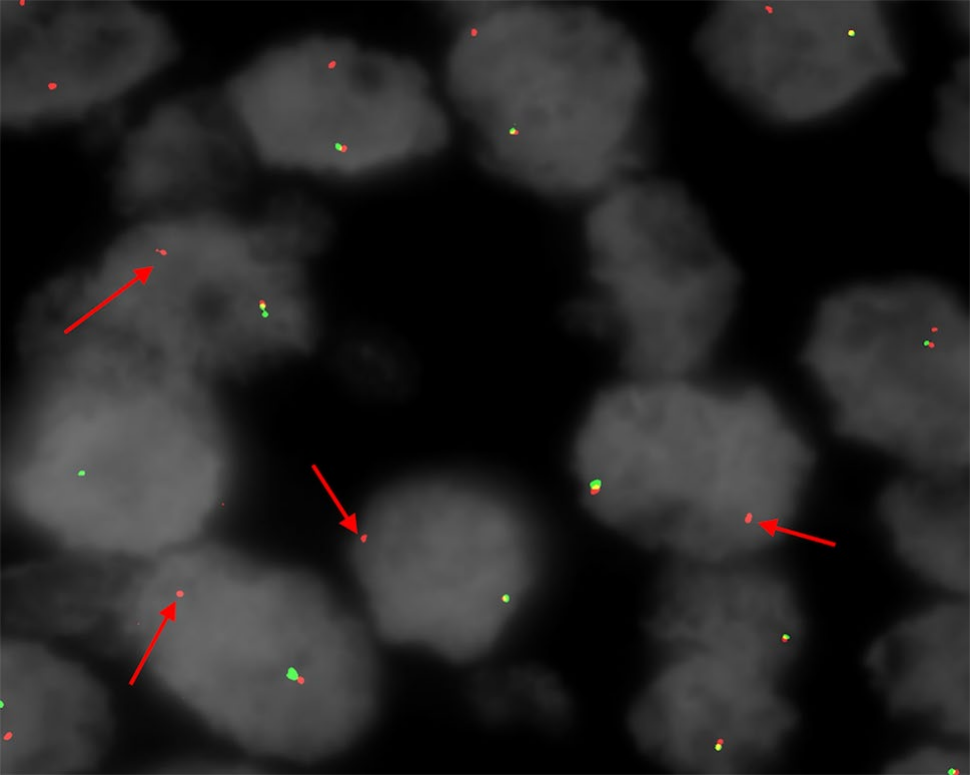
*NUTM1* break-apart fluorescence in situ hybridization (FISH) result showing loss of 5′*NUTM1* signal (green fluorophore) and retention of 3′*NUTM1* signal (red fluorophore, arrows) consistent with rearrangement (Mayo Clinic Laboratories *NUTM1* FISH assay; ~ 600 ×)

The *NUTM1* break-apart probe FISH test was positive for rearrangement of the *NUTM1* locus (Fig. 5). (FISH was performed as an alternate confirmatory method of the NGS results.)

## Discussion

This case is the first reported of a thyroid carcinoma exhibiting thyrocyte differentiation and an *NSD3::NUTM1* fusion. In contrast to all other reported cases of NUT carcinomas harboring a *NUTM1*::bromodomain complex gene fusion, our patient’s tumor showed histologic and immunophenotypic features of a specific, non-squamous carcinoma: (1) thyrocyte differentiation with prominent colloid production, (2) diffuse expression of PAX8 (monoclonal) and TTF1 with thyroglobulin reactivity in colloid, and (3) essentially absent (0 to < 2%) expression of p63, p40, and cytokeratins 5/6. The unequivocal thyroid features by histology and immunophenotype suggest this carcinoma is better classified as a high-grade thyroid carcinoma than a variant NUT carcinoma.

Identification of this histologically unusual tumor as a *NUTM1* fusion thyroid carcinoma required recognition of the histology as unusual, perfoming a panel of IHC antibody stains, and following up initial negative genetic test results with extended molecular genetic testing. We pursued additional, expanded molecular testing on the resected tumor because of the puzzling absence of specific thyroid carcinoma molecular alterations in the Afirma Xpression Atlas™ test performed on the preceding cytology specimen. Importantly, some commercial molecular genetic panels that are configured specifically for the identification or exclusion of thyroid malignancy do not contain *NUTM1* fusions, including the Afirma Xpression Atlas™ assay (refer to the Afirma website for the list of alterations covered). In a case of a thyroid carcinoma with unusual morphology and absence of *BRAF*^V600E^ and *RAS* mutations (assessed by IHC or targeted genetic testing), a broader NGS panel that includes capacity to detect *NUTM1* fusions may be considered in the workup of the tumor.

NUT carcinoma is mechanistically driven by the oncogenic activity of the fused NUT protein and a bromodomain complex (BDC) protein [9]. Histologically, these *NUTM1*::bromodomain complex gene fusion tumors comprise a poorly differentiated or undifferentiated squamous carcinoma (SC), and approximately 33% exhibit mature squamous differentiation described as commonly “abrupt” [10]. Very few NUT carcinomas exhibit histology discordance from poorly differentiated to undifferentiated SC. These cases with non-classical histology include tumors that exhibit strands of cells embedded in a chondromyxoid matrix resembling myoepithelial carcinoma, basaloid tumors with mucin production and cribriforming pattern akin to basaloid SC, and tumors composed of rhabdoid cells or small round cells [3, 5, 11]. Importantly, for these reported non-classical cases, the tumors usually demonstrated an immunophenotype classic for NUT carcinoma: diffuse p63 and or p40 expression and speckled nuclear expression of NUT antibody in greater than 50% of tumor cells [3, 5]. However, recently, two primary lung *BRD4::NUTM1* fusion carcinomas were reported that showed dual histology with areas typical of NUT carcinoma as well as foci of micropapillary carcinoma. Both cases diffusely expressed p63 and NUT consistent with classic NUT carcinoma, but one case also showed patchy cytokeratin 7, Napsin-A, and TTF1 expression in the micropapillary component, consistent with some divergent adenocarcinoma differentiation [12].

Cytologically, our patient’s tumor comprised cohesive groups distinguishing it from other reports of cytologic features of NUT carcinoma which emphasize cellular dyscohesion [13–15]. Nuclear grooves, as found in our case, have been described in one cytology report of a lung *BRD3::NUTM1* fusion tumor, but nuclear pseudo-inclusions observed in our case on the cytology specimen have not been reported in NUT carcinomas [15]. Moreover, the scant viscous colloid globules found on cytology smears in our case are unique. Squamous differentiation was absent in our case but has been described in some NUT carcinoma cytology specimens [13, 14]. Evidence of squamous differentiation in these other cases comprised dyskeratotic cells, keratin, and squamous pearls [13, 14].

Histologically, at initial diagnostic evaluation, the closest conventional fit for this tumor was poorly differentiated thyroid carcinoma (PDTC). Like PDTC, the tumor exhibited increased mitotic activity and necrosis consistent with the Memorial Sloan Kettering Cancer Center criteria for PDTC. It also conformed to the Turin criteria for PDTC with a solid growth pattern, as well as the increased mitoses and necrosis. However, three features of the tumor were diffusely found and distinctively odd for PDTC: (1) perivascular swirling arrangement of tumor cells around peculiar, micro-proliferated vessels (vessels such as seen in glioblastoma); (2) abundant colloid distributed predominantly as extracellular globules consistent with poorly formed follicles; and (3) squamoid appearance of most tumor cells but with sparse, intermingled cells showing nuclear features suggestive of papillary thyroid carcinoma. PDTC is often associated with a well-differentiated carcinoma such as papillary or follicular carcinoma, but such a component was absent in our patient’s tumor. Other diagnoses were also entertained. Anaplastic thyroid carcinoma was diagnostically excluded because of the colloid production, only mild nuclear pleomorphism, and diffuse TTF1 and PAX8 reactivity. Secretory carcinoma was considered due to the droplet-like distribution of the eosinophilic globules, but the immunophenotype, including thyroglobulin expression in the globules, excluded the diagnosis. Cribriform morular carcinoma also entered the diagnostic differential because of the distinct nodularity of the tumor, but the tumor lacked a cribriform architecture and contained colloid which is typically absent in cribriform morular carcinoma.

While the patient’s tumor showed a degree of cytologic atypia and mitotic activity compatible with but not sufficient in themselves for a diagnosis of PDTC, the molecular genetic findings also distinguished it from most cases of poorly differentiated thyroid carcinoma. Approximately 85% of PDTC are postulated to arise from a well-differentiated thyroid carcinoma that has accrued additional genetic alterations in a stepwise molecular progression [8, 16]. These 85% of poorly differentiated thyroid carcinomas not only contain *BRAF*^*V600E*^ or *RAS* mutations as found in well-differentiated carcinomas but also show additional mutations in the *TERT* promoter, *TP53*, PIK3CA pathway genes, and/or others [8, 16–20]. However, up to 14% of PDTC contain gene fusions (involving *RET*, *ALK*, and *PPARG*), but no *NUTM1* fusion has been described [8, 16, 17].

Three other cases of NUT carcinomas arising in the thyroid have been reported (see Table 2) [6–8]. Two cases were diagnosed as NUT carcinoma and one as anaplastic thyroid carcinoma. In distinct contrast to our case, two cases showed the classic histology of NUT carcinoma [6, 7], while the third case lacks evaluable histologic details and was diagnosed as anaplastic thyroid carcinoma [8]. The case lacking histologic details occurred in a 34-year-old woman and was included in a molecular genetic study of poorly differentiated and anaplastic thyroid carcinomas (*n* = 84 and 33, respectively) [8]. No details were provided of either the histologic features or immunophenotype (see Table 2, case 3). The first case arose in a 42-year-old woman and exhibited classic features of NUT carcinoma including monomorphic cells with abrupt squamous differentiation (see Table 2, case 1) [6]. Tumor cells diffusely expressed NUT protein and p63. The second case occurred in a 34-year-old man (see Table 2, case 2) [7]. The post-chemotherapy resected tumor comprised a pleomorphic carcinoma with extensive squamous differentiation. Tumor cells expressed TTF1, PAX8, and p63, NUT protein expression was “equivocal,” and there was no expression of thyroglobulin. A biopsy that preceded neoadjuvant chemotherapy was described as typical of NUT carcinoma comprising a monomorphic population of cells with abrupt keratinization. Notably, the fusion found in the 2016 reported case was *BRD4::NUTM1*, while an *NSD3::NUTM1* fusion was identified in the other two cases [6–8].

**Table 2:** Comparison of four reported cases of NUT fusion carcinomas occurring in the thyroid gland

**Age-Sex**	**Histology**	**IHC**	**Fusion**	**Tumor Extent**	**Treatment**	**Outcome**	**Reference**
42-F	Poorly differentiated Ca with abrupt squamous differentiation Background of chronic lymphocytic thyroiditis and prior SMECE	Positive: p63 and NUT Ab (both diffuse)	*NSD3::NUTM1* (Exon 7 of *NSD3* breakpoint chr8:38,184,248 to Exon 3 of *NUTM1* breakpoint chr15:34,640,166)	Large tumor centered in prior thyroidectomy bed with encasement of trachea	Complete resection Local irradiation Cisplatin Etoposide Molibresib, a BETi (limited course)	Alive with NED 18 months post diagnosis of NUT Ca	Agaimy et al. [6]
34-M	Poorly differentiated Ca with abrupt squamous differentiation on initial biopsy; pleomorphic Ca with squamous differentiation on resection after neo-adjuvant therapy	Positive: p63, TTF1 and PAX8 Negative: thyroglobulin, BRAFV600E Equivocal: NUT Ab	*NSD3::NUTM1*	Large, multinodular thyroid tumor with gross extrathyroidal extension; 5 of 59 lymph nodes positive and mediastinal lymph node metastases	Complete resection Local irradiation Cisplatin Pembrolizumab	Alive with NED 38 months postop	Kuo et al. [7]
34-F	Anaplastic thyroid Ca with foci of poorly differentiated thyroid Ca (no further details provided)	Not provided	*NUTM1::BRD4* (Exons 1–2 of *NUTM1* and Exons 14–20 of *BRD4*)	Not provided; estimated to be high stage (T3b or T4) based on surgical procedures	Complete resection Local irradiation	Alive 120 months post diagnosis	Landa et al. [8]
72-F	High grade CA with mixed squamoid and PTC-like cells, poorly formed follicles and colloid without keratinization Background of mild chronic lymphocytic thyroiditis and multinodular hyperplasia	Positive: PAX8, TTF1, thyroglobulin, keratin 7 Negative: p63 and p40 (2% only), cytokeratins 5/6, BRAF^V600E^ Equivocal: NUT antibody	*NSD3::NUTM1* fusion (Exon 7 breakpoint of *NSD3* and Exon 3 breakpoint of *NUTM1*; transcript ID NM_017778.2/NM_175741.2)	Large multinodular thyroid tumor with gross extrathyroidal extension involving carotid sheath; negative for lymph node metastases; negative for distant metastases	Complete resection Radioactive iodine ablation	Alive with NED at 3 months post diagnosis	Current patient

*F* female, *M* male, *IHC* immunohistochemistry, *SMECE* sclerosing mucoepidermoid carcinoma with eosinophilia, *BETi* bromodomain and extraterminal domain protein small molecular inhibitor, *Ca* carcinoma, *NED* no evidence of disease, *PTC* papillary thyroid carcinoma

Thus, at the molecular genetic level, three of the now four reported cases of primary thyroid carcinomas with *NUTM1* fusions, including our case, contained the *NSD3* fusion partner. NSD3 is a histone methyltransferase that binds to the extraterminal (ET) domain of bromodomain-extraterminal domain (BET) proteins [21]. The BET proteins, together with NSD3, p300, and others, normally form an epigenetically functional bromodomain complex (BDC) that attaches to specific chromatin sites and acetylates histones allowing transcription to proceed [4]. Oncogenic compound NUT-bromodomain complex proteins, including the *NSD3::NUTM1* protein, create a “massive super enhancer” that forces sustained, markedly increased transcription of specific genes including *MYC*, *SOX2*, and *p63* while repressing transcription of various tumor suppressor genes [9, 13]. The *NSD3::NUTM1* fusion protein functions essentially analogously to the more common *BRD4/BRD3::NUTM1* fusion protein, but minor differences may be present [5, 22]. Indeed, one minor difference comprises occasional lack of or equivocal NUT protein expression on immunohistochemical staining, which is consistent with what occurred in our case [5, 7].

An oncogenic feature of NUT carcinomas also found in some thyroid carcinomas is MYC protein overexpression [4, 23, 24]. Thyroid carcinomas arising through the effects of MYC overexpression include some papillary carcinomas, follicular carcinomas, poorly differentiated carcinomas, and anaplastic carcinomas [25, 26]. In thyroid carcinomas, MYC overexpression develops by various means, including *MYC* amplification or rearrangements, *BRAF*^*v600e*^ mutation interactions, specific long noncoding RNA dysregulation, increased BRD4 protein production, and abnormal TERT expression, among others [25, 27–31]. This patient’s tumor may represent another means (e.g., *NUTM1* fusion) by which MYC overexpression compels oncogenesis in the neoplastic thyrocyte.

In summary, we present an *NSD3::NUTM1* fusion thyroid carcinoma clearly showing thyrocyte differentiation, including colloid production and poorly formed follicles. This tumor may represent a hitherto unrecognized group of *NUTM1* fusion-positive, high-grade thyroid carcinomas that exhibit distinctive features of swirling nodular growth of squamoid cells around micro-proliferated blood vessels, prominent colloid production, nuclear features overlapping with papillary thyroid carcinoma in an intermingled subset of tumor cells, and a thyrocyte immunophenotype. The predicted outcome of this carcinoma is unknown; the pT3 AJCC stage and vascular invasion at presentation suggest it could behave aggressively. Although the three other reported patients with *NUTM1* fusion thyroid carcinomas experienced prolonged survival, of the two cases with sufficient details to evaluate, the patients had histologically and/or immunophenotypically typical NUT carcinoma, and moreover, all three were significantly younger than our patient. Should our patient’s tumor prove an index case of a specific, high-grade, thyrocyte-differentiated, *NUTM1* fusion thyroid carcinoma, discovery of more examples will establish the range of pathology features, tumor behavior, and response to therapies such as BETi agents and immune checkpoint inhibitors.

## Data Availability

Available upon request.
